# Crosstalk between skeletal muscle ratio and cholesterol metabolism disorders: a cross-section study

**DOI:** 10.1186/s12902-024-01660-y

**Published:** 2024-07-24

**Authors:** Yunle Wang, Jun Hu, Hui Shen, Chunxing Liu, Lijuan Yang

**Affiliations:** 1 Geriatrics Department, Shanghai Health and Medical Center, 67 Dajishan, Wuxi, Jiangsu, 214000 China; 2 Health Care Center, Shanghai Health and Medical Center, Wuxi, China; 3 Nutritional Department, Shanghai Health and Medical Center, Wuxi, China; 4 Medical Laboratory Department, Shanghai Health and Medical Center, Wuxi, China

**Keywords:** Body composition analysis, Skeletal muscle index, Skeletal muscle mass ratio, BMI, Hypercholesterolemia

## Abstract

**Background:**

Dysfunction of cholesterol metabolism may be associated with low skeletal muscle mass.  This study aimed to explore the relationship between skeletal muscle mass and cholesterol metabolic disorders in adults.

**Methods:**

The data of a total of 5949 people with complete medical history data, biochemical data and body composition analysis were recruited. According to the serum cholesterol, low density lipoprotein (LDL), high density lipoprotein (HDL) and nonHDL, the population was divided into a disorder group and a normal group. Independent sample t tests, chi-square tests, Pearson's correlation analyses and binary logistic regression analyses were used to study the effect of body composition on abnormal cholesterol metabolism. According to BMI and sex, the population was divided into different subgroups, and binary logistic regression analysis was used to study the effect of the skeletal mass ratio on cholesteral metabolic disorders in different subgroups.

**Results:**

There were significant differences in sex, alcohol consumption, body weight, BMI, skeletal muscle mass index (SMI) [total skeletal muscle mass (kg)/height 2 (m2)] and skeletal muscle mass ratio (SMR) [total skeletal muscle mass (kg)/weight (kg) *100] between the disorder group (hypercholesterolemia, hyper-LDL, lower-HDL and hyper-nonHDL) and the normal group. Pearson correlation analysis revealed that the SMR was negatively correlated, while the SMI was positively correlated with cholesterol metabolic disorders in both sexes. The overweight group was older and had a greater SMI, abnormal cholesteral metabolism ratio and lower SMR than the normal-weight group. In the normal-weight group, the SMR was an independent protective factor against different kinds of cholesteral metabolic disorders in both sexes, while the SMI was a risk factor. In the overweight subgroup, the protective effect on HDL and nonHDL metabolism remained in the male subgroup but disappeared in the female subgroup. However, the SMI was an independent risk factor for different kinds of cholesteral metabolic disorders in both sexes.

**Conclusions:**

SMR was an independent protective factor against cholesterol metabolic disorders in both males and females, especially in the normal weight group. SMI was an independent risk factor, especially in the overweight group.

## Introduction

Serum cholesterol and its lipoprotein carriers (low density lipoprotein [LDL], very low-density lipoprotein [VLDL], and high density lipoprotein [HDL]) are known to be related to ASCVD. Hypercholesterolemia is an independent risk factor for atherosclerotic cardiovascular disease (ASCVD) [1]. In China, the number of patients with ASCVD and hypercholesterolemia is increasing, and the age of onset is becoming younger, which may be related to genetics, modern diet, work and rest patterns [2, 3]. Current treatments for hypercholesterolemia include lifestyle modification and drug therapy [4]. However, drug therapy has side effects [5], some of which can be severe. How to reduce serum cholesterol by physiological means has become an important issue.

Emerging research suggests that exercise could modulate cholesteral metabolism mainly in obese adults [6] or those with metabolic disorder syndromes such as diabetes [7]. Recent studies have shown that skeletal muscle also has an important effect on metabolic disorders. A decrease in skeletal muscle mass may be associated with significant metabolic consequences for older adults [8]. Associations between skeletal muscle mass and metabolic syndrome [9, 10], insulin resistance [10], and inflammation [11] have been reported. Several investigations have suggested that low skeletal muscle mass increases the risk of metabolic syndrome [10] and a metabolically obese phenotype over time [12]. These results prove that skeletal muscle is involved in metabolism and that muscle rain may improve metabolic disorders.

At present, there is little research on skeletal muscle evaluation indices related to cholesterol metabolism. Some studies have shown that the use of CT to detect abdominal muscle density is associated with metabolic abnormalities; however, CT is not the first choice for daily monitoring [13]. Body composition analysis is widely used in health examinations and has no side effects. The skeletal muscle index (SMI), a muscle index calculated by body composition analysis, is widely used to evaluate muscle mass in individuals with sarcopenia. However, the protective effect of SMI in metabolic dysregulation diseases remains to be discussed [14]. The study found that obese people generally had higher SMI than the normal population. The skeletal muscle involved in regulating lipid metabolism results are not consistent. It may be that SMI is not an effective measure of skeletal muscle mass. In this study, we used the skeletal muscle rate as an index of evaluation, which has rarely been mentioned in cholesterol metabolic disorder studies, and found that it has a protective effect on cholesterol metabolism in the Chinese population.

## Methods

### Study design and data collection

Eighteen- to sixty-year-old adults who underwent medical examinations at the Shanghai Medical Care Center from 2022.01 to 2023.09 were included. There were 3698 males and 2466 females who underwent body composition analysis. Patients who had a full medical history and blood examination results during the same period were included. Subjects with hypertension, diabetes, or the use of lipid-lowering drugs were excluded from the study. Subjects which taking medications that could affect body weight or body composition were excluded. After excluding ineligible patients, 3551 males and 2398 females were included in the analyses. Ethics approval and consent to participate: This study was performed in accordance with the principles of the Declaration of Helsinki and was approved by the Ethics and Research Committee of Shanghai Medical Care Center (NO. 2024–04). Informed consent was waived by our Institutional Review Board because of the retrospective nature of our study.

### Definitions and diagnostic criteria

The diagnostic criteria of hypercholesterolemia was serum cholesterol $\ge$ 5.20mmol/l, hyper-LDL was LDL $\ge$ 3.37mmol/l, low-HDL was HDL $\le$ 1.04mmol/l and hyper-nonHDL was nonHDL $\ge$ 4.2mmol/l. As weight and BMI may be collinear with the SMI and SMR, we did not include BMI or weight in the multivariable logistic regression. We divided the population into different subgroups according to BMI. Due to the differences in body size, the overweight standard for Chinese people was BMI $\ge$ 24kg/m^2^ and normal weight standard was 18.5 $\le$ BMI $<$ 24.

### Body composition analysis

A body composition analyser (InBody 770) which use BIA to measure weight, fat mass, and skeletal muscle mass. The skeletal muscle mass index [SMI = total skeletal muscle mass (kg)/height^2^ (m^2^)] and skeletal muscle mass ratio [SMR (%) = total skeletal muscle mass (kg)/weight (kg) *100] were obtained.

### Clinical and laboratory measurements

All blood samples were obtained in the morning after a 12-h overnight fast for subsequent assays. Serum total cholesterol was determined enzymatically using a chemistry analyser (Roche cobras c702).

### Statistical analysis

The data are expressed as the mean $\pm$ SD, median or percentage. Differences between groups were tested using Student’s t test, and the $\chi$ ^2^ test was used to test for differences in the distribution of categorical variables. Each variable was examined for a normal distribution. A correlation analysis of the SMI with other metabolic variables was conducted. Odds ratios (ORs) and 95% confidence intervals (CIs). *P* < 0.05 was considered to indicate statistical significance in all analyses. All the statistical results were based on two-sided tests. The data were analysed using SPSS (R27.0.0.0) for Mac.

## Results

### Characteristics of the subjects

The characteristics of the patients’ basic data are presented in Table 1. There were 3551 males and 2398 females, and the mean age was 43.6 years. The hypercholesterol ratio was 38.4%, the hyper-LDL ratio was 36.6%, the low-HDL ratio was 17.8%, and the hypernonHDL ratio was 24.3%.

**Table 1:** Characteristics of the subjects

Characteristic	Mean (SD)/Percent (n)
Sex (female)	40.3 (2398)
Age (Y)	43.61 $\pm$ 10.76
Alcohol consumption	42.9 (2553)
Exercise	51.2 (3045)
Weight (kg)	68.66 $\pm$ 12.89
BMI (kg/m^2^)	24.21 $\pm$ 3.35
SMI (kg/m^2^)	9.53 $\pm$ 1.41
SMR	39.48 $\pm$ 3.83
Hyper-TC	38.4 (2284)
Hyper-LDL	36.6 (2175)
Low-HDL	17.8 (1056)
Hyper-nonHDL	24.3 (1444)

### Characteristics of the normal cholesteral and disordered cholesteral groups

The characteristics of the groups are presented in Table 2. The subjects in the cholesteremia disorder group had greater weight, higher BMI, higher SMI, higher alcohol consumption rate and lower SMR.

**Table 2:** Characteristics of the normal cholesteral and disordered cholesteral groups

	Sex (female, %)	Age	Alcohol (%)	Exercise	Weight	BMI	SMI	SMR
normal-TC	40.9	42.3 $\pm$ 10.9	41.6%	50.5%	68.2 $\pm$ 13.0	24.0 $\pm$ 3.4	9.5 $\pm$ 1.4	39.7 $\pm$ 3.8
hyper-TC	39.4	45.6 $\pm$ 10.2	45.1%	52.3%	69.4 $\pm$ 12.7	24.5 $\pm$ 3.3	9.6 $\pm$ 1.4	39.2 $\pm$ 3.8
*P* ($\text{\rm X}$ ^2^)	0.13	$<$ 0.01	0.01	0.10	$<$ 0.01	$<$ 0.01	$<$ 0.01	$<$ 0.01
normal-LDL	43.3	42.6 $\pm$ 11.0	41.0%	50.9%	67.6 $\pm$ 13.0	23.9 $\pm$ 3.4	9.4 $\pm$ 1.4	39.6 $\pm$ 3.8
hyper-LDL	35.1	45.4 $\pm$ 10.1	46.2%	51.7%	70.5 $\pm$ 12.5	24.8 $\pm$ 3.2	9.7 $\pm$ 1.4	39.3 $\pm$ 3.8
*P* ($\text{\rm X}$ ^2^)	$<$ 0.01	$<$ 0.01	$<$ 0.01	0.29	$<$ 0.01	$<$ 0.01	$<$ 0.01	0.04
normal-HDL	45.0	43.7 $\pm$ 11.0	40.8%	51.2%	67.1 $\pm$ 12.5	23.9 $\pm$ 3.2	9.4 $\pm$ 1.4	39.4 $\pm$ 3.9
Low-HDL	18.4	43.2 $\pm$ 9.7	57.1%	51.1%	75.6 $\pm$ 12.6	25.9 $\pm$ 3.3	10.3 $\pm$ 1.3	39.8 $\pm$ 3.4
*P* ($\text{\rm X}$ ^2^)	$<$ 0.01	0.26	$<$ 0.01	0.50	$<$ 0.01	$<$ 0.01	$<$ 0.01	$<$ 0.01
normal-nonHDL	44.7	42.8 $\pm$ 10.9	40.5%	51.1%	67.4 $\pm$ 12.8	23.9 $\pm$ 3.3	9.4 $\pm$ 1.4	39.5 $\pm$ 3.9
hyper-nonHDL	26.6	46.1 $\pm$ 10.0	50.3%	51.3%	72.5 $\pm$ 12.4	25.3 $\pm$ 3.2	10.0 $\pm$ 1.3	39.4 $\pm$ 3.7
*P* ($\text{\rm X}$ ^2^)	$<$ 0.01	$<$ 0.01	$<$ 0.01	0.45	$<$ 0.01	$<$ 0.01	$<$ 0.01	0.60

### Correlation of the SMI and SMR with cholesteral

Table 3 shows the correlation analysis of several continuous variables and different cholesteral compositions after stratification by sex. Age, weight, BMI and SMI were positively related to LDL and nonHDL but negatively related to HDL in the female group. In male group BMI was positively related to cholesterol, and nonHDL but negatively related to HDL. Additionally, the SMR was negatively related to cholesterol, LDL and non-HDL but positively related to HDL in both sexes.

**Table 3:** Correlation of the SMI and SMR with cholesteral

	TC	LDL	HDL	nonHDL
Female				
Age	*r* = 0.31 *P* $<$ 0.01	*r* = 0.32 *P* $<$ 0.01	*r* = -0.10 *P* $<$ 0.01	*r* = 0.35 *P* $<$ 0.01
Weight	*r* = 0.04 *P* = 0.05	*r* = 0.11 *P* $<$ 0.01	*r* = -0.31 *P* $<$ 0.01	*r* = 0.17 *P* $<$ 0.01
BMI	*r* = 0.08 *P* $<$ 0.01	*r* = 0.17 *P* $<$ 0.01	*r* = -0.37 *P* $<$ 0.01	*r* = 0.24 *P* $<$ 0.01
SMI	*r* = 0.01 *P* = 0.51	*r* = 0.07 *P* $<$ 0.01	*r* = -0.27 *P* $<$ 0.01	*r* = 0.12 *P* $<$ 0.01
SMR	*r* = -0.12 *P* $<$ 0.01	*r* = -0.18 *P* $<$ 0.01	*r* = 0.27 *P* $<$ 0.01	*r* = -0.23 *P* = 0.01
Male				
Age	*r* = 0.04 *P* = 0.01	*r* = 0.01 *P* = 0.63	*r* = -0.01 *P* = 0.69	*r* = 0.04 *P* = 0.01
Weight	*r* = 0.03 *P* = 0.07	*r* = 0.01 *P* = 0.55	*r* = -0.30 *P* $<$ 0.01	*r* = 0.12 *P* $<$ 0.01
BMI	*r* = 0.07 *P* $<$ 0.01	*r* = 0.04 *P* = 0.02	*r* = -0.34 *P* $<$ 0.01	*r* = 0.17 *P* $<$ 0.01
SMI	*r* = 0.03 *P* = 0.06	*r* = -0.00 *P* = 0.82	*r* = -0.21 *P* $<$ 0.01	*r* = 0.09 *P* $<$ 0.01
SMR	*r* = -0.09 *P* $<$ 0.01	*r* = -0.08 *P* $<$ 0.01	*r* = 0.32 *P* $<$ 0.01	*r* = -0.18 *P* $<$ 0.01

### Characteristics of the overweight and normal weight groups in both sexes

As weight and BMI may be collinear with the SMI and SMR, we did not include BMI or weight in the multivariable logistic regression. We divided the population into different subgroups according to sex and BMI. The characteristics of the overweight and normal weight groups according to sex are presented in Table 4. In the female and male subgroups, the participants in the overweight group were older (40.69 ± 10.69 vs. 44.95 ± 11.01, *P* $<$ 0.01), had a greater SMI (7.92 ± 0.62 vs. 8.96 ± 0.70, *P* $<$ 0.01), had a greater cholesteral metabolic disorder ratio (hypercholesteral: 25.2% vs. 32.6%, *P* $<$ 0.01, hyper-LDL: 24.2% vs. 36.0%, *P* $<$ 0.01, low-HDL: 26.4% vs. 65.3%, *P* $<$ 0.01, hypernonHDL: 25.6% vs. 40.4%,* P* $<$ 0.01) and had a lower SMR (37.64 ± 2.71 vs. 34.10 ± 2.45, *P* $<$ 0.01). Additionally, in the male subgroup, the overweight subgroup had a greater alcohol consumption rate (60.8% vs. 65.5%, *P* $<$ 0.01).

**Table 4:** Characteristics of the overweight and normal weight groups in both sexes

	Female			Male		
	Mean (SD)/Percent (n)		*P* ($\text{\rm X}$ ^2^)	Mean (SD)/Percent (n)		*P* ($\text{\rm X}$ ^2^)
	Normal weight	Overweight		Normal weight	Overweight	
Number	1753	645		1273	2278	
Age, Y	40.69 $\pm$ 10.69	44.95 $\pm$ 11.01	$<$ 0.01	43.99 $\pm$ 10.98	45.18 $\pm$ 10.18	$<$ 0.01
Alcohol	27.4%	32.4%	0.08	60.8%	65.5%	0.01
Exercise	28.4%	27.5%	0.616	52.7%	54.8%	0.24
SMI, kg/m^2^	7.92 $\pm$ 0.62	8.96 $\pm$ 0.70	$<$ 0.01	9.62 $\pm$ 0.69	10.83 $\pm$ 0.83	$<$ 0.01
SMR	37.64 $\pm$ 2.71	34.10 $\pm$ 2.45	$<$ 0.01	43.37 $\pm$ 2.62	40.35 $\pm$ 2.66	$<$ 0.01
Hyper-TC	25.2%	32.6%	$<$ 0.01	33.9%	41.7%	$<$ 0.01
Hyper-LDL	24.2%	36.0%	$<$ 0.01	35.3%	42.1%	$<$ 0.01
Low-HDL	26.4%	65.3%	$<$ 0.01	13.1%	31.4%	$<$ 0.01
Hyper-nonHDL	25.6%	40.4%	$<$ 0.01	22.2%	29.9%	$<$ 0.01

### Dual-logistic regression analysis for hypercholesteremia

**Fig. 1:**
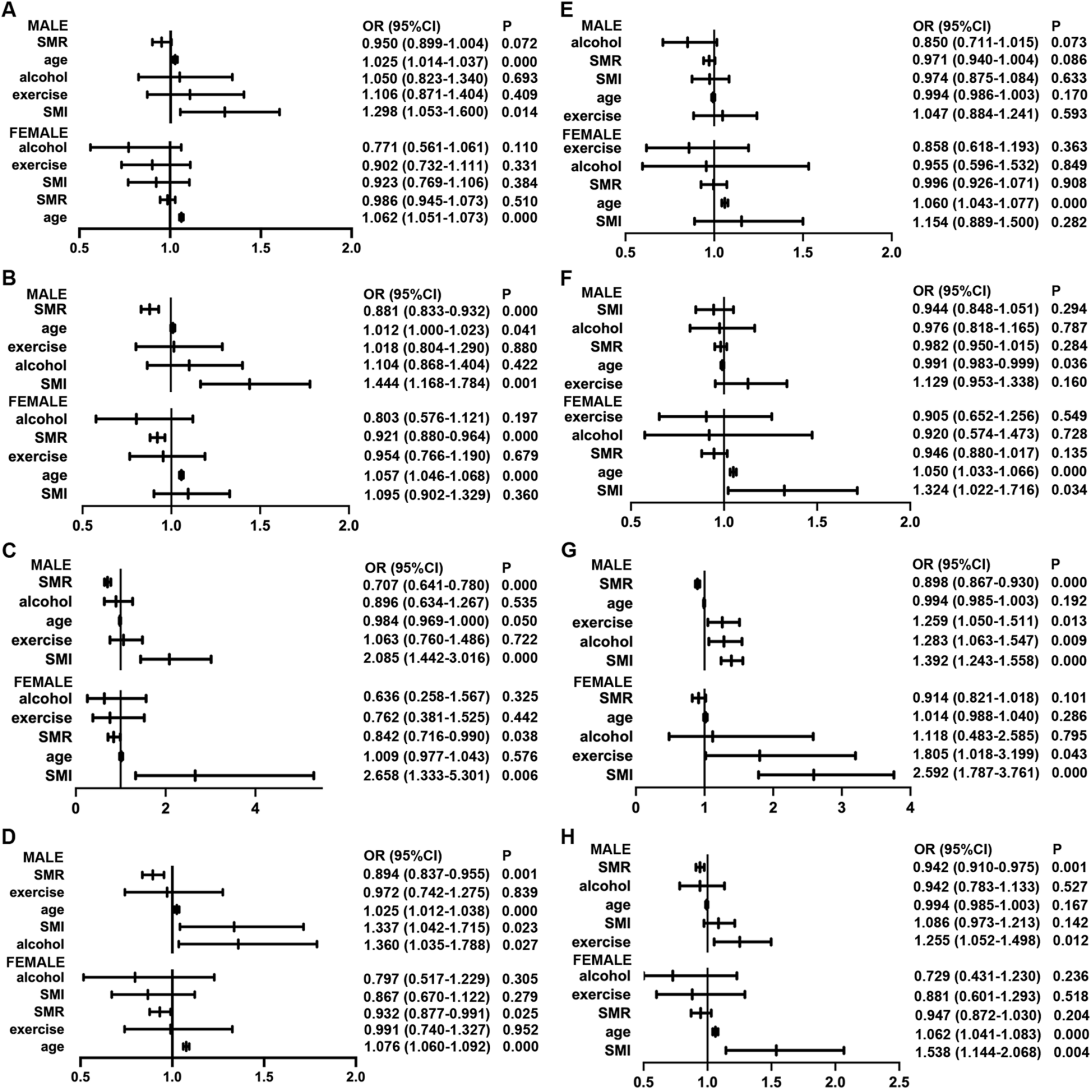
Forester map of logistic regression results. **A** Logistic regression results in the normal-weight group with hyper-TC. **B** Logistic analysis of hyper-LDL levels in the normal-weight group. **C** Logistic analysis of low-HDL-C levels in the normal-weight group. **D** Logistic analysis of hypernon-HDL-C in the normal-weight group. **E** Logistic results in the overweight group with hyper-TC. **F** Logistic results in the hyper-LDL overweight group. **G** Logistic results in the overweight group with low HDL. **H** logistic results in the overweight group of hypernon-HDL

Dual-logistic regression analysis was performed with cholesteral metabolic disorder as the dependent variable, and the ORs and 95% CIs of other variables, including age, alcohol consumption, exercise, SMR and SMI, were calculated (Fig. 1). In the normal-weight subgroup, SMR was an independent protective factor for cholesteral metabolic disorders in female subjects (hyper-LDL: OR = 0.921, 95% CI = 0.880–0.964; low-HDL: OR = 0.842, 95% CI = 0.716–0.990; hypernonHDL: OR = 0.932, 95% CI = 0.877–0.991; Fig. 1B-D) and in male subjects (hyper-LDL: OR = 0.881, 95% CI = 0.883–0.932; low-HDL: OR = 0.707, 95% CI = 0.641–0.780; hypernonHDL: OR = 0.894, 95% CI = 0.837–0.955; Fig. 1B-D). SMI was an independent risk factor in males (hyper-TC: OR = 1.298, 95% CI = 1.053–1.600; hyper-LDL: OR = 1.444, 95% CI = 1.168–1.784; low-HDL: OR = 2.085, 95% CI = 1.442–3.016; hypernonHDL: OR = 1.337, 95% CI = 1.042–1.715; Fig. 1A-D). However, the SMI was an independent risk factor for females with only low-HDL disorders (OR = 2.658, 95% CI = 1.333–5.301; Fig. 1C). Additionally, age was an independent risk factor for all kinds of cholesteral metabolic disorders in both sexes (Fig. 1A-D).

In the overweight subgroup, SMR was an independent protective factor for HDL and nonHDL metabolism in males (low-HDL: OR = 0.898, 95% CI = 0.867–0.930; hypernonHDL: OR = 0.942, 95% CI = 0.910–0.975; Fig. 1G, H), while SMI was an independent risk factor for HDL metabolism (low-HDL: OR = 1.392, 95% CI = 1.243–1.558). In the female subgroup, the SMI was an independent risk factor for cholesteral metabolism disorder (hyper-LDL: OR = 1.324, 95% CI = 1.022–1.716; low-HDL: OR = 2.592, 95% CI = 1.787–3.761; hypernonHDL: OR = 1.538, 95% CI = 1.144–2.068; Fig. 1F-H), while an independent protective effect of SMR did not exist. Age was still an independent risk factor for different kinds of cholesteral metabolic disorders in both sexes (Fig. 1E-H).

## Discussion

In this cross-sectional analysis, we found that cholesterol metabolic disorders were associated with skeletal muscle mass. We used the skeletal muscle rate (SMR, %) = total skeletal muscle mass (kg)/weight (kg) *100 as an indicator of skeletal muscle mass and found that it was an independent protective factor against cholesterol metabolic disorders, especially in normal weight subjects. However, the SMI (skeletal muscle index, kg/m^2^) = total skeletal muscle mass (kg)/height^2^ (m^2^) seemed to be an independent risk factor. Overall, our findings have clinical relevance and suggest that changes in the skeletal muscle rate may contribute to lower levels of serum cholesterol and the risk of metabolic disease.

Current research on exercise and health improvement has focused mainly on weight loss [15, 16]; some people lose weight through dieting but still have lipid metabolism disorders or steatohepatitis [17]. In this study, we found that in cholesterol metabolic disorder groups, hyper-TC, hyper-LDL, low-HDL and high-nonHDL subjects with a normal BMI also had hypercholesterolemia, and these people had a lower proportion of skeletal muscle. These findings demonstrated that skeletal muscle improvement plays an important role in the regulation of lipid metabolism disorders. Several studies have shown that adipose tissue is necessary for the development and regeneration of normal muscle mass and strength [18, 19]. Brown adipose tissue can be activated by myokines secreted by skeletal muscle tissue after exercise [20]. These studies revealed a relationship between lipid metabolism and skeletal muscle.

The SM/height^2^ (SMI) has been widely used to assess sarcopenia [21, 22]. However, in the study of metabolic disorders, the SMI was not a protective factor, as it was positively correlated with glucose and lipid metabolism disorders [14]. In our study, the SMI was greater in the cholesteral disorder groups and was positively correlated with cholesteral metabolism disorders. In both sex subgroups, the SMI was significantly greater in the overweight group than normal weight group. In the normal-weight group, the SMI was an independent risk factor in the male subgroup, while in the overweight subgroup, the effect only existed in the low-HDL subgroup. In the female subgroup, the SMI was an independent risk factor according to the low-HDL analysis in the normal-weight group, while in the overweight group, it was an independent risk factor according to the hyper-LDL, low-HDL and hypernonHDL analyses. This may be because of skeletal muscle hypertrophy, as the SMI was significantly greater in the overweight group [23, 24]. Studies have used X-rays to detect muscle mass and density, which supports this hypothesis [13].

Unlike the SMI, the SMR was proven to be a protective factor against cholesterol metabolic disorders and could be used to measure lipid metabolic disorders. The SMR was an independent protective factor against different kinds of cholesterol metabolic disorders in both the female and male subgroups of normal-weight subjects. In the female subgroup, the SMR reduced the incidence of hyper-LDL by 7.9%, the incidence of low-HDL by 15.8% and the incidence of hypernonHDL by 6.8% in normal-weight subjects. In the male subgroup, the protective effect of SMR was even greater (11.9% decrease in the incidence of hyper-LDL, 29.3% decrease in the incidence of low-HDL, and 10.6% decrease in the incidence of hypernonHDL). Additionally, in overweight subjects, the protective effect of SMR existed in the male subgroup (10.2% decrease in the incidence of low HDL, 5.8% decrease in the incidence of hypernonHDL), while in the female subgroup, no independent protective effect was found. The results showed that SMR was an independent protective factor against cholesterol metabolic disorders, especially in the normal-weight population, and had a greater protective effect on HDL metabolism.

At present, exercise guidance for blood lipid regulation in hypercholesterolaemia patients is still mainly based on weight loss [25]. Research on sarcopenia has focused mainly on older and obese people [26, 27], with fewer studies on young and middle-aged people or people of normal weight. In this study, we found that even in normal-weight subjects, there was also a high incidence of hypercholesterolemia. These people were older, had relatively greater BMIs and SMIs and had lower SMRs. These results proved that skeletal muscle was important for cholesteral metabolism, especially in the normal-weight group, and that the SMR was a protective factor against skeletal muscle mass. The exercise recommendation for people with high cholesterol, especially those with normal BMI, should not only be to reduce fat, but also to increase muscle training. It may be more conducive to BMI normal cholesterol metabolism.

## Limitations

The population in this study was a healthy physical examination population, and there was a lack of data on disease population, so the sample could not represent the whole population. May exist in the study did not control other confounding factors, the study on chronic disease poor generalization in the crowd.

## Conclusion

The skeletal muscle rate (SMR) and skeletal muscle index (SMI) were associated with the incidence of cholesterol metabolic disorders, and the SMR was an independent protective factor, while the SMI was an independent risk factor. The protective effect of SMR existed mainly in the normal-weight group, and it had the most significant effect on HDL metabolism.

## Data Availability

Data is provided within the manuscript.
